# Germline genome editing versus preimplantation genetic diagnosis: Is there a case in favour of germline interventions?

**DOI:** 10.1111/bioe.12635

**Published:** 2019-08-25

**Authors:** Robert Ranisch

**Affiliations:** ^1^ Institute of Ethics and History of Medicine, University of Tübingen Germany

**Keywords:** assisted reproduction, CRISPR, germline gene therapy, germline genome editing, preimplantation genetic diagnosis, non-identity problem

## Abstract

CRISPR is widely considered to be a disruptive technology. However, when it comes to the most controversial topic, germline genome editing (GGE), there is no consensus on whether this technology has any substantial advantages over existing procedures such as embryo selection after in vitro fertilization (IVF) and preimplantation genetic diagnosis (PGD). Answering this question, however, is crucial for evaluating whether the pursuit of further research and development on GGE is justified. This paper explores the question from both a clinical and a moral viewpoint, namely whether GGE has any advantages over existing technologies of selective reproduction and whether GGE could complement or even replace them. In a first step, I review an argument of extended applicability. The paper confirms that there are some scenarios in which only germline intervention allows couples to have (biologically related) healthy offspring, because selection will not avoid disease. In a second step, I examine possible moral arguments in favour of genetic modification, namely that GGE could save some embryos and that GGE would provide certain benefits for a future person that PGD does not. Both arguments for GGE have limitations. With regard to the extended applicability of GGE, however, a weak case in favour of GGE should still be made.

## 1 INTRODUCTION

For a long time, direct interventions into the human germline were widely seen as a red line that should not be crossed.1
For discussions on germline interventions before the advent of genome editing technologies, see e.g. Lappé, M. (1991). Ethical issues in manipulating the human germ line. *Journal of Medicine and Philosophy, 16*(6), 621–639; Wivel, N., & Walters, L. (1993). Germ‐line gene modification and disease prevention: Some medical and ethical perspectives. *Science, 262*(5133), 533–538; Stock, G., & Campbell, J. (2000). *Engineering the human germline. An exploration of the science and ethics of altering the genes we pass to our children*. Oxford, U.K.: Oxford University Press.
 This could change following the development of new tools for gene editing such as CRISPR/Cas9. Recently, germline genome editing (GGE) has been applied in various animal models and was used in primates in 2014. A year later, a Chinese team published data from the first experiments on (non‐viable) human embryos.2
Liang, P., Xu, Y., Zhang, X., Ding, C., Huang, R., Zhang, Z., … Huang, J. (2015). CRISPR/Cas9‐mediated gene editing in human tripronuclear zygotes. *Protein & Cell, 6*(5), 363–372.10.1007/s13238-015-0153-5PMC441767425894090
 Since then, a handful of studies have reported experiments with gene editing techniques such as CRISPR or base editors in preimplantation human embryos. GGE has been used to correct mutations associated with the blood disease beta‐thalassemia, heart disease and other disorders. GGE was also applied to create mutations in embryos in vitro that are associated with resistance against HIV.3
Kang, X., He, W., Huang, Y., Yu, Q., Chen, Y., Gao, X., … Fan, Y. (2016). Introducing precise genetic modifications into human 3PN embryos by CRISPR/Cas‐mediated genome editing. *Journal of Assisted Reproduction and Genetics, 33*(5), 581–588.10.1007/s10815-016-0710-8PMC487044927052831
 In November 2018, a Chinese scientist claimed to have created the world's first genetically edited babies.4
Cyranoski, D., & Ledford, H. (2018). International outcry over genome‐edited baby claim. *Nature*, *563*(7733), 607–608; Lovell‐Badge, R. (2019). CRISPR babies: A view from the centre of the storm. *Development, 146*, dev175778.10.1038/d41586-018-07545-030482929



In principle, different strategies can be used to target the germline genome.5
Vassena, R., Heindryckx, B., Peco, R., Pennings, G., Raya, A., Sermon, K., & Veiga, A. (2016). Genome engineering through CRISPR/Cas9 technology in the human germline and pluripotent stem cells. *Human Reproduction Update, 22*(4), 411–419.10.1093/humupd/dmw00526932460
 First, editing systems can be injected into the human zygote, leading to genetically modified embryos. Second, gene editing can be employed in human germline cells (sperm or eggs) or their progenitors. Third, gene modifications can be applied to pluripotent stem cells, which could then be grown into gametes and be used for fertilization. This paper focuses on the first approach of GGE.

Because of its inefficiency in introducing genetic changes, GGE is still seen as too risky for human reproduction. Gene editing tools sometimes cut non‐targeted genes, leading to off‐target effects, or do not reach all cells, causing mosaicism in embryos.6
Hershlag, A., & Bristow, S. L. (2018). Editing the human genome: Where ART and science intersect*. Journal of Assisted Reproduction and Genetics*, *35*(8), 1367–1370; Kang, X. J., Caparas, C. I. N., Soh, B. S., & Fan, Y. (2017). Addressing challenges in the clinical applications associated with CRISPR/Cas9 technology and ethical questions to prevent its misuse. *Protein & Cell, 8*(11), 792–794.10.1007/s13238-017-0477-4PMC567659628986765
 Off‐target effects or mosaicism have been detected in most experiments on human embryos.7
Hershlag et al., op. cit. note 6.
 Thus, in likely clinical scenarios for GGE, the outcome of modifications would have to be controlled by means of genetic diagnosis before modified embryos could be transferred. Critically, genetic testing cannot detect off‐target mutations reliably and does not allow testing for mosaicism.8
Vassena et al., op. cit. note 5, p. 413.



The most promising results from introducing genetic changes in embryos to date were published in 2017.9
Ma, H., Marti‐Gutierrez, N., Park, S. W., Wu, J., Lee, Y., Suzuki, K., … Mitalipov, S. (2017). Correction of a pathogenic gene mutation in human embryos. *Nature, 548*, 413–419.10.1038/nature2330528783728
 A mutation was targeted that is associated with a heart disease, seemingly with a high efficiency and a low rate of mosaicism. The experiments have been considered as marking a shift to a possible clinical application of GGE. Certain conclusions of this trial, however, are still contested and have led to an ongoing debate over the appropriate interpretation of the results from this experiment.10
Egli, D., Zuccaro, M., Kosicki, M., Church, G., Bradley, A., & Jasin, M. (2018). Inter‐homologue repair in fertilized human eggs? *Nature, 560*, E5–E7.10.1038/s41586-018-0379-530089924



Current developments are being received ambivalently and have already produced an extensive literature on the ethics of GGE.11
van Dijke, I., Bosch, L., Bredenoord, A. L., Cornel, M., Repping, S., & Hendriks, S. (2018). The ethics of clinical applications of germline genome modification: A systematic review of reasons. *Human Reproduction, 33*(9), 1777–1796.10.1093/humrep/dey257PMC645446730085071
 Following the alleged birth of gene‐edited babies, the question for many experts is not whether GGE will be used in clinical trials for human reproduction, but when. Some argue that such research could allow the development of precise therapies to cure inherited diseases or reduce the risk of passing on genetic dispositions for various disorders. Prominent voices openly promote pushing germline editing into medical practice.12
Daley, G. Q., Lovell‐Badge, R., & Steffann, J. (2019). After the storm—A responsible path for genome editing. *The New England Journal of Medicine, 380* (10), 897–899; Savulescu, J., & Singer, P. (2019). An ethical pathway for gene editing. *Bioethics, 33* (2), 221–222; Church, G. (2015). Perspective: Encourage the innovators. *Nature, 528*, S7.10.1056/NEJMp190050430649993
 The issue of genetic enhancement is also being discussed, but is far from becoming a reality.13
Gyngell, C., Bowman‐Smart, H., & Savulescu, J. (2019). Moral reasons to edit the human genome: Picking up from the Nuffield report. *Journal of Medical Ethics.* 10.1136/medethics-2018-105084PMC682014730679191



Conversely, other voices point to the unprecedented risks of introducing irreversible mutations into the human genome. Because genetic modifications can be passed on to subsequent generations, it is hard to contain possible side‐effects. Apart from safety concerns, further arguments against GGE call attention to the lack of consent from future offspring, possible eugenic or slippery slope effects, possible resulting social inequalities, and concerns about ‘designer babies’.14
van Dijke et al., *op. cit.* note 11.



Germline interventions are prohibited under various national legislations15
Isasi, R., Kleiderman, E., & Knoppers, B. M. (2016). Editing policy to fit the genome? *Science, 351*(6271), 337–339.10.1126/science.aad677826797999
 as well as under the UNESCO Universal Declaration on the Human Genome and Human Rights. The so‐called Oviedo Convention allows genetic intervention ‘only if its aim is not to introduce any modification in the genome of any descendants’ (Section 13). Nevertheless, the 2017 report on ‘Human genome editing: Science, ethics, and governance’ from the U.S. National Academies of Sciences, Engineering, and Medicine (NASEM) suggested that clinical research and application using GGE should be permitted, and that in some scenarios GGE ‘would provide the only or the most acceptable option for parents who desire to have genetically related children’.16
National Academies of Sciences, Engineering, and Medicine. (2017). *Human genome editing: Science, ethics, and governance.* Washington, DC: The National Academies Press, p. 102.28796468
 This conclusion was considered a paradigm shift towards the acceptability of GGE.17
Baylis, F. (2017). Human germline genome editing and broad societal consensus. *Nature Human Behaviour, 1*(6).



Despite expressing a favourable opinion, the NASEM do not embrace GGE unconditionally. Clinical trials (as well as future application) are deemed to be permissible only under certain conditions, for example if ‘data on risks and potential health benefits of the procedures’ are available and interventions are restricted to ‘preventing a serious disease’. First and foremost, the NASEM stress that future trials are only legitimate in ‘the absence of reasonable alternatives’.18
NASEM, op. cit. note 16, p. 6.
 A similar constraint has been discussed by other institutions such as the Nuffield Council.19
Nuffield Council on Bioethics. (2016). Genome editing: An ethical review. Retrieved from http://nuffieldbioethics.org/wp-content/uploads/Genome-editing-an-ethic-alreview.pdf, p. 46; Nuffield Council on Bioethics. (2018). Genome editing and human reproduction. Retrieved from http://nuffieldbioethics.org/wp-content/uploads/Genome-editing-and-human-reproduction-FINAL-website.pdf, pp. 20–22.
 Considering the uncertainties and possible risks associated with GGE, it is imperative to employ less risky technologies if they can achieve the same ends. Evitt and colleagues, who were among the first to develop a regulatory framework for trials on GGE, argue in a similar vein: clinical research may only happen when the effects of GGE cannot be achieved by established techniques, in particular by embryo selection and prenatal genetic diagnosis.20
Evitt, N. H., Mascharak, S., & Altman, R. B. (2015). Human germline CRISPR‐Cas modification: Toward a regulatory framework. *American Journal of Bioethics, 15*(12), p. 26.10.1080/15265161.2015.1104160PMC469947726632357



This paper will explore whether and under which conditions GGE could have any advantage over existing technologies. The focus will be on a comparison between GGE as a type of genetic intervention or modification and embryo selection after preimplantation genetic diagnosis (PGD). This technology of selective reproduction has been widely discussed as a major alternative to germline modification. However, opinions diverge significantly over possible advantages and disadvantages. Regarding the possibility of embryo selection, some authors see no real benefit of GGE over existing methods such as PGD and thus consider genetic interventions as superfluous.21
Lander, E. S. (2015). Brave new genome. *New England Journal of Medicine, 373*(1), 5–8; Lanphier, E., Urnov, F., Haecker, S. E., Werner, M., & Smolenski, J. (2015). Don't edit the human germ line. *Nature, 519*, 410–411; Lundberg, A. S., & Novak, R. (2015). CRISPR‐Cas gene editing to cure serious diseases: Treat the patient, not the germ line. *American Journal of Bioethics, 15*(12), 38–40; Mertes, H. & Pennings, G. (2015). Modification of the embryo’s genome: More useful in research than in the clinic. *American Journal of Bioethics, 15*(12), 52–53.; Hershlag et al., op. cit. note 6; Hildt, E. (2016). Human germline interventions–think first. *Frontiers in Genetics, 7* (81).
 Others point to the limitations of PGD and highlight that these could be overcome in the future by GGE.22
de Wert, G., Heindryckx, B., Pennings, G., Clarke, A., Eichenlaub‐Ritter, U., van El, C. G., …. Cornel, M. C. (2018). Responsible innovation in human germline gene editing. Background document to the recommendations of ESHG and ESHRE. *European Journal of Human Genetics, 26*(4), 450–470; Porteus, M. H., & Dann, C. T. (2015). Genome editing of the germline: Broadening the discussion. *Molecular Therapy, 23*(6), 920–980; Steffann, J., Jouannet, P., Bonnefont, J. P., Chneiweiss, H., & Frydman, N. (2018). Could failure in preimplantation genetic diagnosis justify editing the human embryo genome? *Cell Stem Cell, 22*(4), 481–482; Vassena et al., op. cit. note 5.10.1038/s41431-017-0077-zPMC589150229326429
 In addition, some consider GGE to be the morally better strategy,23
Gyngell, C., Douglas, T., & Savulescu, J. (2017). The ethics of germline gene editing. *Journal of Applied Philosophy, 34*(4), 498–513; Cavaliere, G. (2018). Genome editing and assisted reproduction: Curing embryos, society or prospective parents? *Medicine, Health Care and Philosophy, 21*(2), 215–225.
 some see it as morally equal to PGD and embryo selection,24
Shaw, J. (2018). Selecting for disabilities: Selection versus modification. *New Bioethics*, *24*(1), 44–56.10.1080/20502877.2018.144167129529983
 while others reject GGE in favour of selection.25
Rehmann‐Sutter, C. (2018). Why human germline editing is more problematic than selecting between embryos: Ethically considering intergenerational relationships. *New Bioethics*, *24*(1), 9–25.10.1080/20502877.2018.144166929529985



## 2 WHAT ARE THE ALTERNATIVES?

The demand for GGE is most likely to arise in situations where one or both reproducers are a known carrier or sufferer of a genetic disease and want to prevent the transmission of the disease‐causing mutation to their offspring. Thus, the desire to have the chance of conceiving a healthy child drives the development and possible application of this new technique.

It follows that, regarding the permissibility of GGE, it must be asked what can count as a reasonable alternative for intended parents. Arguing that some couples should simply refrain from reproduction would not qualify as such, because this option precludes the desired end, namely starting a family. It must rather be asked what means allow intended parents to have healthy offspring. In this context, the attributed value of having biologically related offspring is a decisive factor. After all, sperm or egg donation, surrogacy, and adoption could allow couples to have a healthy child. But the preference for genetic over non‐genetic parenthood is widespread.26
Hendriks, S., Peeraer, K., Bos, H., Repping, S., & Dancet, E. A. F. (2017). The importance of genetic parenthood for infertile men and women. *Human Reproduction, 32*(10), 2076–2087.10.1093/humrep/dex25628938731
 While there might be pragmatic and perhaps moral reasons to change this preference (e.g. in relation to the possible benefits for orphans), the question of the (il)legitimate moral weight of the desire to have a genetic link to one’s offspring will not be discussed in this paper. Rather, the common wish for biological relatedness will be taken for granted here, and the focus will lie on existing and future technological means that could help couples to have biological children.

### 2.1 Somatic gene therapy

In the context of a disease‐carrying couple, two alternatives to GGE are frequently proposed: therapy after birth, and selective reproduction. Developments of gene editing technologies such as CRISPR, zinc finger nucleases (ZFNs) or TALENs give rise to new approaches for somatic gene therapies. Experiments in animal models and preclinical studies27
Maeder, M. L., & Gersbach, C. A. (2016). Genome‐editing technologies for gene and cell therapy*. Molecular Therapy, 24*(3), 430–446.10.1038/mt.2016.10PMC478692326755333
 suggest promising applications, and the first human trials are getting underway, for example for different types of cancer. Experimental treatments using TALEN gene‐edited T‐cells have been used in infants, indicating the therapeutic potential of gene editing technologies.28
Qasim, W., Zhan, H., Samarasinghe, S., Adams, S., Amrolia, P., Stafford, S., … Veys, P. (2017). Molecular remission of infant B‐ALL after infusion of universal TALEN gene‐edited CAR T cells. *Science Translational Medicine, 9* (374).10.1126/scitranslmed.aaj201328123068
 Various trials have begun that focus on inherited disorders such as beta‐thalassemia and hemophilia.29
Porteus, M. H. (2019). A new class of medicines through DNA editing. *New England Journal of Medicine, 380* (10), 947–959.10.1056/NEJMra180072930855744
 This could lead to novel treatments that may render some applications of GGE obsolete in the future.30
Lundberg et al., op. cit. note 21.



However, somatic gene therapy would not be an efficient alternative to GGE in all cases. Even though new therapies could, in principle, be used to correct the specific somatic cells of a newborn or a child and ameliorate the condition, some congenital or early‐onset diseases would affect a subject severely before any therapy was feasible. In some cases, for example forms of lysosomal storage disorders, newborns show symptoms by the first days of life and do not survive infancy. In the case of Duchenne muscular dystrophy (DMD), which often manifests around the age of three to five, degenerative effects are almost impossible to reverse after symptoms appear. DMD and other disorders also affect widespread and different types of tissues, making it difficult for a somatic therapy to reach all affected cells.31
NASEM, op. cit. note 16, p. 88.
 Moreover, tissues in which the genetic disease is manifest are sometimes hard to access, for example in neurodegenerative disorders such as Huntington’s disease.32
Porteus et al., op. cit. note 22, p. 981.
 When a couple is a known carrier of such mutations, GGE on an early embryo could be advantageous over somatic gene therapy after birth, because only single cells of gametes or zygotes would need to be targeted in vitro. Compared with this, for somatic gene therapy to be effective, a large number of cells would need to be targeted. Furthermore, in the context of GGE, possible therapeutic failures can be contained better, because embryo selection or even abortion remains an option.33
Gyngell et al., op. cit. note 23, p. 505.



In addition, successful GGE would have a multi‐generational advantage over somatic therapies. Even though GGE cannot eradicate inherited diseases for good, because offspring may develop new mutations or mate with a carrier, GGE could reduce the frequency of mutations in future generations. In summary, while somatic gene therapy could become an alternative to GGE for specific pathologies, direct intervention into the human germline would likely be the more effective strategy in some cases.

### 2.2 Selective reproduction

Selective reproduction is commonly proposed as another alternative means that allows couples to have healthy, biological offspring. Selective reproduction encompasses various attempts ‘to create one possible future child rather than a different possible future child’.34
Wilkinson, S. (2010). *Choosing tomorrow’s children: The ethics of selective reproduction.* Oxford, UK: Oxford University Press, p. 2.
 This includes invasive or non‐invasive procedures of prenatal testing, which could lead to the selective termination of the pregnancy. Because abortion is most often invasive and stressful for women, and the moral status of a fetal life is widely considered to be higher than that of the human embryo, selection after preimplantation genetic diagnosis (PGD) must be seen as the preferable alternative to abortion.

Just like most scenarios for germline therapies, PGD presupposes assisted reproduction (e.g. IVF or intracytoplasmic sperm injection). Preimplantation embryos are then analysed for genetic mutations, and only unaffected embryo(s) are transferred. Although assisted reproduction is considered to be safe, hormone stimulation, egg retrieval procedures, and low success rates often put physical, mental and financial burdens on the woman.

Within certain limitations, PGD is permitted under various regulatory regimes.35
Isasi et al., op. cit. note 15.
 In the U.K., for example, under the regulations of the Human Fertilisation and Embryology Authority, PGD is allowed for more than 500 conditions. In addition to the main application, namely the avoidance of single‐gene disorders such as cystic fibrosis, PGD and embryo selection have been used to avoid chromosomal aberrations, to reduce genetic risks (e.g. for breast cancer), for sex selection, and for HLA typing. Some companies claim to be able to screen for some polygenic conditions,36
Genomic Prediction. Retrieved from https://genomicprediction.com/faqs/

 and, controversially, fertility clinics began offering PGD to select for cosmetic traits such as the eye colour of the future child.37
The Fertility Institute. Choose Your Baby’s Eye Color. Retrieved from https://www.fertility-docs.com/programs-and-services/pgd-screening/choose-your-babys-eye-color.php




Owing to its wide applicability and its favourable risk profile, PGD is frequently considered to be the major alternative to GGE.38
Lander, op. cit. note 21, p. 6; Lanphier et al., op. cit. note 21, p. 411.
 If PGD and embryo selection could be used to achieve the same end as direct modification, there seems to be little or no justification for further research and for the development of GGE techniques for human reproduction. Thus, GGE and PGD need to be compared regarding the possible advantages of germline intervention over selection. With this in mind, two related topics will be analysed: the possible clinical (Section 3) and moral (Section 4) advantages of GGE over PGD.

## 3 THE LIMITS OF PGD AND THE CLINICAL ADVANTAGE OF GGE

While PGD can sometimes give intended parents the chance to have healthy offspring, it is not an effective strategy in all cases.39
Nuffield Council on Bioethics (2016), op. cit. note 19, p. 46; Nuffield Council on Bioethics (2018), op. cit. note 19, pp. 44–45; Gyngell et al., op. cit. note 23; NASEM, op. cit. note 16, pp. 86–88; Lovell‐Badge, op. cit. note 4.
 There are scenarios in which PGD will always be useless or where the chances are significantly low that selective reproduction can help intended parents to have a child that does not carry the mutation. In addition, the transfer of unaffected embryos may be feasible, but couples might object to the means or ends of selective reproduction.

The clearest cases where PGD is futile are occasions where a would‐be parent is homozygous for an autosomal‐dominant disease (e.g. Huntington´s disease or Marfan syndrome) or where both parents are homozygous for an autosomal‐recessive disease (e.g. cystic fibrosis). In such cases, it is impossible not to pass on a mutated allele to any future offspring. The same is true for a parent that has a chromosomal aberration in germline cells due to homologous Robertsonian translocation (e.g. leading to Translocation Down syndrome in offspring).

The case of inherited mitochondrial diseases is special. These are often severe diseases with a high variability in symptoms that can be caused by mutations in the maternal mitochondrial DNA (mtDNA). Although PGD has been used to prevent the transmission of mitochondrial disorders, usually this is not possible. For homoplasmic mutations of mtDNA, every copy of mtDNA carries a deleterious mutation (e.g. leading to possible manifestation of Leber´s hereditary optic neuropathy in offspring).40
Bredenoord, A. L., Pennings, G., Smeets, H. J., & De Wert, G. M. W. R. (2007). Dealing with uncertainties: Ethics of prenatal diagnosis and preimplantation genetic diagnosis to prevent mitochondrial disorders*. Human Reproduction Update, 14*(1), 83–94.10.1093/humupd/dmm03718056133
 When mutant and normal mtDNA occur together (heteroplasmy), embryos can be affected to different degrees (e.g. leading to Leigh syndrome or MELAS in offspring). Here, PGD could only be used to identify those with the lowest mutation load. Mitochondrial replacement therapy (MRT) has been used for human reproduction, although only in a few cases.41
Zhang, J., Liu, H., Luo, S., Chavez‐Badiola, A., Liu, Z., Yang, M., … Huang, T. (2016). First live birth using human oocytes reconstituted by spindle nuclear transfer for mitochondrial DNA mutation causing Leigh syndrome. *Fertility and Sterility, 106*(3), 375–376.
 This procedure to avoid the occurrence of inherited mitochondrial diseases, however, involves third‐party egg cell donors. Future developments of GGE could make this superfluous. Genome editing tools such as CRISPR could be used here to target mitochondria for editing,42
Jo, A., Ham, S., Lee, G. H., Lee, Y. I., Kim, S., Lee, Y. S., … Lee, Y. (2015). Efficient mitochondrial genome editing by CRISPR/Cas9. BioMed Research International.10.1155/2015/305716PMC458150426448933
 and it has been shown that TALENs could reduce the mutation load.43
Reddy, P., Ocampo, A., Suzuki, K., Luo, J., Bacman, S. R., Williams, S. L., … Izpisua Belmonte, J. C. (2015). Selective elimination of mitochondrial mutations in the germline by genome editing. *Cell, 161*(3), 459–469.10.1016/j.cell.2015.03.051PMC450583725910206



Whether these cases provide a reasonable ground to justify research and development for GGE is contentious. It is frequently stressed that situations where PGD is not useful are extremely rare, suggesting that GGE would still not be acceptable even if it were the only effective treatment in the future.44
Lander, op. cit. note 21; Mertes et al., op. cit. note 21; Hildt, op. cit. note 21.
 It is estimated that 152 births per year in the U.K. and 778 in the United States involve women with inherited mtDNA mutations.45
Gorman, G. S., Grady, J. P., Ng, Y., Schaefer, A. M., McNally, R. J., Chinnery, P. F., … Turnbull, D. M. (2015). Mitochondrial donation—How many women could benefit? *New England Journal of Medicine, 372*(9), 885–887.10.1056/NEJMc1500960PMC448129525629662
 Homozygous carriers of a dominant disease may suffer from a severe disease, making it unlikely that they reach reproductive age. However, it has been highlighted that better medical treatment prolongs the life of patients and that they sometimes meet each other in support groups and may start a family. Also, the high global number of consanguineous marriages, which increase the risk of transmitting inherited disease, should be considered.46
Church, op. cit. note 12.



Even if the exact numbers of such constellations were so low as to make these cases insignificant compared with other diseases, it is hard to see how this fact alone could make GGE illegitimate and thereby deny some couples the chance of having healthy offspring. The argument of rarity does not provide a reasonable moral justification to ban GGE. On the contrary, when direct intervention into the germline is the only or most reasonable option to have healthy offspring, GGE could increase reproductive options for some couples and thus extend reproductive autonomy.

In addition to scenarios where PGD never gives parents the chance to have healthy offspring, more frequently there are cases where it is unlikely (to various degrees) to be possible to select embryos that would not have the deleterious mutation. When both parents are heterozygous for autosomal‐dominant conditions, on average three out of four embryos will be affected by the condition. In principle, PGD is an option here, but the chances of conceiving a healthy child are low because the number of unaffected embryos is highly reduced. Here more scenarios for application are conceivable, for example Y‐linked gonosomal conditions.47
Nuffield Council on Bioethics (2016), op. cit. note 19, p. 46.



In all these cases, a sufficient number of embryos would usually be needed to allow the transfer of suitable embryos. This, however, is a key challenge in assisted reproduction and PGD. Gyngell and colleagues calculated that, in the U.K. alone, more than 120 IVF cycles are conducted for PGD each year and only produce one single viable embryo.48
Gyngell et al., op. cit. note 13.
 Based on data from a PGD Centre in France, Steffann and colleagues point to a similar problem: viable and morphologically suitable embryos are frequently discarded after genetic profiling, leaving only a small number of unaffected embryos available for transfer.49
Steffann et al., op. cit. note 22.
 In situations like this, GGE could be used in the future. When used as a complementary tool rather than as an alternative for PGD, the editing of embryos could raise the number available for transfer and increase pregnancy rates.50
de Wert et al, op. cit. note 22, pp. 465–466.
 It is estimated that for several hundred couples each year, this might be the only option for having healthy offspring.51
Gyngell et al., op. cit. note 13.
 This strategy could also be beneficial for older women or for women who have had cancer treatment, from whom it can be difficult to retrieve a high number of egg cells.

A compelling case in favour of GGE can also be made with regard to polygenic conditions.52
Savulescu, J., Pugh, J., Douglas, T., & Gyngell, C. (2015). The moral imperative to continue gene editing research on human embryos. *Protein & Cell, 6*(7), 476–479; Gyngell et al., op. cit. note 13, pp. 501‐502.10.1007/s13238-015-0184-yPMC449105026113289
 Even though there are a few thousand known monogenetic diseases, they only make up a fraction of the global burden of disease.53
World Health Organization. Genes and human disease. Retrieved from http://www.who.int/genomics/public/geneticdiseases/en/index2.html

 Most diseases that have a genetic component are polygenic, including some forms of cancer, diabetes, or coronary artery disease. Here it is not a single mutation that leads to the manifestation of the disease. The joint contribution of various genetic and environmental factors contributes to an increased risk. Despite a growing interest in using PGD for polygenic conditions, such selection is hardly feasible, because an enormous number of embryos would be needed to find the preferred genotype. In theory, GGE could be used to change various gene loci directly and decrease the susceptibility for multifactorial conditions.

This advantage of GGE over PGD is, however, still speculative. While CRISPR has been used in animal models to modify more than one gene locus, this comes with additional risk. Editing several targets at the genome increases the likelihood of off‐target effects and other adverse effects on the developing embryo. This might be overcome by future developments.54
See, e.g., Zhang, H., Pan, H., Zhou, C., Wei, Y., Ying, W., Li, S., … Ding, X. (2018). Simultaneous zygotic inactivation of multiple genes in mouse through CRISPR/Cas9‐mediated base editing. *Development, 145* (20), dev168906.10.1242/dev.16890630275281
 But still, not much is known about dispositions that depend on variants in many gene loci. While future genome‐wide association studies could reveal some genetic risk factors and improve our understanding of polygenic conditions, it remains unclear to what extent and hence whether these insights could be used for germline therapy.55
Tam, V., Patel, N., Turcotte, M., Bossé, Y., Paré, G., & Meyre, D. (2019). Benefits and limitations of genome‐wide association studies. *Nature Reviews Genetics*, 20(8), 467–48410.1038/s41576-019-0127-131068683



Beyond avoidance of disorders in future offspring, GGE might be used by parents to have a child which is compatible as cell or organ donor for a diseased sibling. Selection is not always possible to conceive such a ‘savior sibling’. Then GGE could, in principle, allow the genetic modification of an otherwise healthy embryo to produce a matching child.56
Lovell‐Badge, R. (2019), op. cit. note 4.
 However, a more likely scenario enables the use of donor stem cells that have been modified to serve the same purpose.

There are also conceivable scenarios where intended parents have idiosyncratic reproductive desires, for example for non‐medical traits such as eye colour, that could not be realized by embryo selection. Almost all non‐medical traits of interest such as intelligence are likely to depend on multiple genetic (and environmental) factors.57
Shulman, C., & Bostrom, N. (2014). embryo selection for cognitive enhancement: Curiosity or game‐changer? *Global Policy, 5*(1), 85–92.
 Quite apart from the moral issues involved in such forms of genetic enhancement, it is still not known whether GGE could be used in such applications58
Janssens, A. C. (2016). Designing babies through gene editing: Science or science fiction? *Genetics in Medicine, 18*(12), 1186–1187.10.1038/gim.2016.2827054708



Finally, one could imagine couples who could have healthy offspring by using assisted reproduction but who reject selective reproduction on religious or moral grounds. For the first live birth following MRT, it was reported that the woman was motivated by religious reasons to undergo the highly experimental procedure of spindle nuclear transfer.59
Zhang et al., op. cit. note 41, p. 376.
 In a similar way, GGE might seem an attractive option for some intended parents because it could avoid the destruction of embryos after PGD and offer a direct fix for affected embryos. But, as will be discussed in the next section, such a scenario seems highly unlikely.

In view of all the above, the widely held claim that there is no clinical advantage of GGE ‘over existing and developing methods’60
Lanphier, op. cit. note 21, p. 411.
 does not hold true. Even though embryo selection after PGD often allows monogenetic disorders to be avoided, it is not a feasible strategy in all constellations. Sometimes direct modification could provide the only possible way to give intended parents the chance to have healthy biologically related offspring. Notably, this conclusion about the extended applicability of germline editing is a factual not a moral statement. The normative implications depend on additional considerations. For now, however, it is safe to conclude that a prima facie case in favour of GGE can be made. Following the recommendations from the NASEM and others, in consideration of the lack of alternatives, GGE could be a legitimate option for certain cases.

## 4 THE MORAL ADVANTAGE OF GGE

Apart from the extended applicability of germline modification, several sources have proposed that GGE has moral advantages over embryo selection after PGD. While GGE and selective reproduction can both be used to give intended parents the chance of having healthy offspring, different strategies are employed. In the case of selection, only embryos with a suitable genotype are transferred to the uterus after genetic testing. Embryos with genetic anomalies or surplus embryos are normally discarded. By contrast, GGE could at least in theory ‘repair’ affected embryos,61
Genome editing: Science, ethics, and public engagement. *Lancet, 390*, 625.10.1016/S0140-6736(17)32209-228816123
 which makes it a unique tool for interventions in preimplantation embryos. This advantage is expressed in two distinct arguments in favour of GGE: the argument of embryo protection and the argument of benefit.

### 4.1 The argument of embryo protection

It is widely believed that human life in its early stage has some value, which constitutes enough reason for embryo protection. This claim is sometimes stated in absolute terms, namely that embryos have the same moral status as adult human beings or persons. More frequently, a moderate version is defended, arguing that human embryos have some value at the very least, which distinguishes them from a mere bunch of cells. These assumptions are echoed in various legislations in which embryo research is prohibited or only allowed under certain restrictions.

The moral status attributed to embryos is also one of the reasons why PGD is controversial. Genetic embryo testing often occurs together with selection, namely the selective transfer of only some cells and the discarding of affected embryos. In the context of reproductive medicine, it is sometimes proposed that GGE would be a morally better option than PGD and selection, because modification could give parents ‘the possibility of rescuing their affected embryos’.62
Steffann et al., op. cit. note 22; de Wert et al., op. cit. note 22, p. 14; Gyngell et al., op. cit. note 23, p. 504.
 By allowing a direct fix, embryos could be transferred that otherwise would have been discarded.

While this outcome would be welcome from the perspective of the moral status of embryos, it is not a likely scenario for most cases. If GGE was considered for preimplantation embryos, those embryos that are affected by gene mutations would need to be identified.63
Hershlag et al., op. cit. note 6, p. 1369.
 In order to not put the ‘healthy’ embryos at risk of superfluous interventions, selection would be performed to identify embryos with mutations that could be targeted for genetic modification. Then, however, it is not clear why the available and unaffected embryos should not be transferred in the first place.64
Lander, op. cit. note 21.
 It would be paradoxical to reject suitable embryos after PGD in order to give embryos that carry a disease‐causing mutation a chance of being cured. Only after failed attempts to transfer unaffected embryos would it seem plausible to edit otherwise unsuitable embryos that are now routinely discarded.65
Steffann et al., op. cit. note 22; de Wert et al., op. cit. note 22, p. 14; Ma et al., op. cit*.* note 9, p. 413.



An additional technical hurdle, which could lead to embryo loss, arises here. Gene editing of the embryo should happen early, ideally before the first cell division, that is, directly with or right after fertilization.66
Winblad, N., & Lanner, F. (2017). At the heart of gene edits in human embryos. *Nature, 548*, 398–400.10.1038/nature2353328783721
 At this point of development, however, PGD is not feasible without destroying the embryo. Thus, when not all embryos are affected, one either has to neglect embryo testing and apply GGE ‘blindly’ at an early stage, thereby risking harming some otherwise suitable non‐mutant embryos, or applying GGE later after mutant embryos were identied and thereby accepting a loss in efficacy owing to an increased risk of mosaicism. Both options create additional risks of embryos being damaged.

Saving embryos is conceivable mainly in those rare instances described above, where it is expected that all embryos from a couple would have disease alleles. But even then, rather than avoiding PGD, GGE would create an additional indication for testing embryos. Owing to risks such as off‐target effects, success in GGE outcome would need to be validated. Even though PGD does not guarantee to detect off‐target effects, in a likely scenario it will still be performed after intervention, to reduce the risk of adverse effects of GGE. Hence, as long as GGE is not perfectly accurate, embryos might again be discarded even after the intervention.

In consequence, it is unlikely that GGE will have a significant effect on rescuing embryos. It is even less likely that GGE will soon become a ‘replacement for PGD’.67
Gyngell et al., op. cit. note 23, p. 504.
 In a possible future scenario of GGE, embryo‐testing and possibly selection will likely be conducted once or even twice: after the intervention and most often before intervention. Thereby embryos might be rejected for transfer either because enough unaffected embryos are available for transfer, or because genetic testing shows that GGE was not successful.

Those who espouse the moral status of embryos can claim a second advantage in favour of GGE. Apart from therapeutic applications, gene editing technologies provide powerful tools for basic research, which could allow new insights into embryonic development or into reasons for infertility.68
Plaza Reyes, A., & Lanner, F. (2017). Towards a CRISPR view of early human development: Applications, limitations and ethical concerns of genome editing in human embryos. *Development, 144*(1), 3–7; Gyngell et al., op. cit. note 23, p. 503.10.1242/dev.13968328049687
 Natural embryo loss is a common phenomenon: implantation failure or spontaneous abortion affect the majority of human embryos.69
Ord, T. (2008). The scourge: Moral implications of natural embryo loss. *American Journal of Bioethics, 8*(7), 12–19.10.1080/1526516080224814618759175
 Approximately one out of six couples experience involuntary childlessness over the period of a year. In the context of assisted reproduction, the baby‐take‐home rate is about 15 percent per IVF cycle, meaning that most fertilized eggs are not viable, do not develop or implant, or are spontaneously aborted.

There are various possible scenarios regarding how developments around genome editing could be beneficial here. Some form of ‘personalized assisted reproduction’70
Ishii, T. (2017). Germ line genome editing in clinics: The approaches, objectives and global society. *Briefings in Functional Genomics, 16*(1), p. 50.10.1093/bfgp/elv053PMC529118926615180
 can be imagined in the future, whereby some genetic conditions that are associated with infertility or miscarriage are corrected, thus increasing the chance that embryos will develop and survive. Insights from basic research could also shed light on the beginning of human life. In 2017, a British research team was the first to use CRISPR/Cas9‐mediated genome editing to investigate genetic factors in the development of human embryos, which could allow ‘improvements … in IVF treatments’71
Fogarty, N. M. E., McCarthy, A., Snijders, K. E., Powell, B. E., Kubikova, N., Blakeley, P., … Niakan, K. K. (2017). Genome editing reveals a role for OCT4 in human embryogenesis. *Nature, 550*, p. 73.10.1038/nature24033PMC581549728953884
 or reduce the number of spontaneous abortions of some human embryos in the future.72
Gyngell et al., op. cit. note 23, p. 504.



From the perspective of embryo protection, it should be noted, however, that basic research with gene editing will often lead to the destruction of embryos. Most research on GGE so far has used triploid (3PN) human embryos, which are believed to be non‐viable,73
In rare cases, 3PN embryos implant and live birth have been documented: Joergensen, M. W., Agerholm, I., Hindkjaer, J., Bolund, L., Sunde, L., Ingerslev, H. J., & Kirkegaard, K. (2014). Altered cleavage patterns in human tripronuclear embryos and their association to fertilization method: A time‐lapse study. *Journal of Assisted Reproduction and Genetics, 31*(4), p. 435.10.1007/s10815-014-0178-3PMC396946424458469
 in order to avoid this moral concern. 3PN embryos, as well as orphan embryos, however, are unsuitable research subjects with which to investigate embryonic development. Thus, research on germline editing itself will likely lead to the creation and destruction of embryos. It has been suggested that this could be justified if genome editing research is likely to reduce global embryo loss in the long run.74
Evitt et al., op. cit. note 20, p. 26.
 This, however, is almost impossible to predict. Insights from experiments with gene editing technology could one day lead to applications that increase the survival rate of embryos, but this is still an open question.

### 4.2 The argument of benefit

Irrespective of the possible protection of embryos, it could be argued that GGE has a real advantage over PGD, because direct interventions might benefit a future person in a way that embryo selection does not. This line of argument has been proposed by Gyngell and colleagues as well as other authors:75
Delaney, J. J. (2011). Possible people, complaints, and the distinction between genetic planning and genetic engineering. *Journal of Medical Ethics, 37*(7), 410–414.10.1136/jme.2010.03942021511971

Genetic selection replaces one individual with a disease with a healthy individual. It does not benefit those with disease. […] GGE on the other hand could provide benefits to individuals who would otherwise be born with genetic disorders – it could cure their disorders.76
Gyngell et al., op. cit. note 23, p. 501.

To put it differently, while GGE could avoid a disease in a future child, PGD and selection avoid a future child with a disease. To illustrate this point, consider two alternative scenarios where a couple has a chance to have healthy offspring using different means:

Case 1: A couple can (a) conceive Ana who carries a genetic disease, or (b) use germline genome editing and conceive Ana who is healthy.

Case 2: A couple can (a) conceive Ana who carries a genetic disease, or (b) use embryo selection and conceive Ben who is healthy.

While in case 1 it can be argued that Ana will exist regardless of the chosen action,77
This claim rests on a few assumptions about the criteria for personal identity, which will not be discussed here. For discussion, see e.g. Parfit, D. (1984). *Reasons and persons.* Oxford, U.K.: Clarendon Press, pp. 352–355; Cavaliere, op. cit. note 23, p. 220.
 the choice in case 2 is identity‐affecting. Depending on the course of action, different people will be born. The person that develops from the embryo choosen after PGD (i.e. Ben), is non‐identical to the person born after natural conception (i.e. Ana)78
Parfit, op. cit*.* note 77, pp. 351–379.



In the first case, a prima facie argument can be made in favour of GGE. By treating the embryo, which Ana develops from, GGE can be regarded as some form of therapy, sometimes described as ‘pre‐emptively therapeutic’.79
Cavaliere, op. cit. note 23, p. 219; Hershlag et al., op. cit. note 6, p. 1369; Rehmann‐Sutter, op. cit. note 25.
 Compared with this, in the second case, PGD has no such therapeutic advantage for the respective child. Either Ana will be born with the genetic disease or she will not exist. Seen in this light, defenders of GGE may claim a therapeutic benefit over PGD.

An obvious problem with this argument arises from such a claim: when it is maintained that it would be better for Ana to be cured by GGE (case 1) than not to be conceived (case 2), why should Ben not be equally considered in this deliberation? Ex hypothesi, he would be born, if the couple decided to use PGD to have healthy offspring. Other things being equal, it is plausible to assume that it would be equally good for Ben to be well and alive as it would be for cured‐Ana. Then, however, it is not clear what the alleged advantage of GGE amounts to, assuming that the omission of GGE leads to the birth of healthy Ben.

To save the argument of therapeutic benefit from this objection, a particular view on values must be maintained: an outcome can only be better (or worse) *for* a particular person. This claim can be described as the *person‐affecting view*. According to this, it cannot be argued that a future person is made better or worse off by an action if the same person’s existence depends on this decision under scrutiny. From this perspective, PGD and selection were not better for Ben in the second case, because otherwise he would not exist. And, conversely, even if sick‐Ana was conceived in this case, she could not complain about her parents’ choice to reject PGD, because otherwise Ana would not exist.80
Delaney, op. cit. note 75.
 This stands in contrast to the first case, where we can imagine sick‐Ana having a legitimate claim against her parents for not being treated with the pre‐emptive therapy.

While the person‐affecting view seems to follow naturally from a commonsense concept of ‘better’ or ‘worse’, another perspective on such valuations is put forward, too. Defenders of an *impersonal view* contend that an outcome can be better (or worse) without being better (or worse) *for* a particular person.81
Smith, K. R., Chan, S., & Harris, J. (2012). Human germline genetic modification: Scientific and bioethical perspectives. *Archives of Medical Research, 43*(7), p. 505.10.1016/j.arcmed.2012.09.00323072719
 Considering the first case, both views come to the same conclusion albeit for different reasons: it is better if the couple decides to use GGE than to forego therapy. According to the impersonal view, GGE leads to a better outcome, for example more health or wellbeing in the world. From the person‐affecting view, GGE is the better option, because it is simply better *for* Ana to be born healthy rather than sick.

In the second case, the two views lead to different conclusions. From the person‐affecting view the choice is morally neutral, since selection would neither be better nor worse *for* sick‐Ana, because otherwise not she, but Ben, would exist (and vice versa). From the impersonal view, it is clearly better to choose PGD and screen out the embryo with the deleterious mutations because only this choice brings about a better state of affairs.

Those who uphold a therapeutic benefit of GGE over PGD are committed to some form of the person‐affecting view. Only if a significant difference can be stated between selection (which ‘replaces’ a future individual) and modification (which can be better for a particular person) can it be maintained that GGE is morally better for someone (here: Ana) compared with PGD. This conclusion cannot be drawn from impersonalism: because the outcomes of PGD and GGE are the same, namely more health in the world, the two options are equally good.

For advocates of the argument of therapeutic benefit, the commitment to some form of personalism comes with a cost. If we accept this view, all identity‐affecting decisions could legitimately neglect the welfare of future persons. If the very existence of future persons depended on the decision under scrutiny, the interest or wellbeing of these future persons would carry no moral weight. Reproductive decisions may then be deeply egocentric, because only the interests of the intended parents (or perhaps of third parties) count. This ‘parentocentric’ view82
Heyd, D. (1992). *Genethics: Moral Issues in the creation of people.* Berkeley: University of California Press, p. 106.
 would render future, possible offspring morally negligible. Whether parents wish to have a healthy or a sick child is not a moral choice, and even the deliberate selection of a sick or disabled child would be morally neutral.83
Bennett, R. (2009). The fallacy of the principle of procreative beneficence. *Bioethics, 23*(5), p. 269.10.1111/j.1467-8519.2008.00655.x18477055
 On the other side, GGE must then be under a totally different regime of regulations: because it could provide a person‐affecting harm or benefit, it can be better or worse for a particular person.

Most defenders of personalism try to mitigate these implications. While they maintain that identity‐affecting decisions usually cannot be worse for a future person, they grant an exception: ‘cases where the child’s life is so awful that we can actually deem it worse than non‐existence’.84
Gavaghan, C. (2007). *Defending the genetic supermarket: Law and ethics of selecting the next generation.* London, U.K.: Routledge‐Cavendish, p. 92; Ibid.
 In situations of a so‐called *wrongful life* it would then be worse if a child was born. According to a different version of this argument, the presumably wrongful life would constitute harm for this child, while it is otherwise impossible to harm by creating life.

This assumption has implications for the evaluation of the second case, where the choice is between PGD (i.e. healthy Ben) and natural conception (i.e. sick‐Ana). If Ana’s genetic disease were so severe as to make her life worse than non‐existence, it would be better not to conceive the embryo from which she developed. Accordingly, choosing PGD would be better in the second scenario, too. This claim seemingly makes it possible to uphold some form of personalism, while not being indifferent to the welfare of future people. However, this deviation from the person‐affecting view is ad hoc.85
Heyd, op. cit. note 82, p. 110.
 If an outcome can only be better (or worse) if it is better (or worse) *for* a person, then even in cases of a horrible life, it cannot be argued that it was better not to exist. Moral limits on reproductive decisions cannot be embraced by the person‐affecting view in this way.

There is a second line of defence, which combines person‐affecting considerations with impersonalism. According to this view, impersonal concerns matter but to a lesser degree than person‐affecting concerns.86
Savulescu, J., Hemsley, M., Newson, A., & Foddy, B. (2006). Behavioural genetics: Why eugenic selection is preferable to enhancement. *Journal of Applied Philosophy, 23*(2), pp. 163–164.10.1111/j.1468-5930.2006.00336.x17036429
 Hence, in the second case it would be worse to conceive sick‐Ana than healthy Ben, but conceiving sick‐Ana in the second case is not as bad as failing to cure Ana in the first case. Even if the outcome is the same, a difference is stated here: while in the second case only a worse state of affairs would have been caused, in the first case a worse state of affairs has been brought about *and* Ana was deprived of being healthy. This claim does not fall into the view that the welfare of certain future people is morally indifferent, while upholding that GGE is in some sense superior to selection.

Such a line of defence, however, suffers from a serious weakness: the proposed benefit of GGE does not only seem to surpass embryo selection. Bringing about a child that has been treated with GGE now seems even more beneficial than bringing about a healthy child. Because then, not only a good state of affairs would be brought about (birth of a healthy child), but also a particular person would have been made better off by being healthy rather than sick. Hence, in the case of a couple that could conceive a healthy child naturally, it would be better to transfer mutant embryos after they were cured.

This implausible implication points to a widely held view on reproductive decisions, or rather on the question of benefit in this context, that is just as paradoxical: while we have reasons to prevent lives from coming into existence, because they would be miserable, we seemingly have no reasons to bring lives into existence, just because they would be happy.87
McMahan, J. (1981). Problems of population theory. *Ethics 92*(1), 96–127.
 In other words, creating a child that has a good life does not seem to be any better in moral terms than not creating a child. This notorious asymmetry is a widely discussed puzzle for any ethics of procreation. However, it poses a special challenge for defenders of the argument of benefit. If it is maintained that GGE is better for a future person compared with PGD it needs to be explained, in what sense bringing a healthy child into existence is beneficial in the first place and, why this is even better than selecting a healthy child.

## 5 CONCLUSION

New tools for genome editing such as CRISPR are widely considered to be disruptive technologies.88
Ledford, H. (2015). CRISPR, the disruptor. *Nature,* 522(7554), 20–24.10.1038/522020a26040877
 But when it comes to the most controversial application, the modification of the human germline, there is no consensus on whether GGE has any real advantages over existing procedures such as embryo selection after PGD. The find an answer to this question is crucial for evaluating whether research, development, and the future application of GGE is legitimate. Considering the risks and uncertainties associated with this new procedure, it has been proposed that GGE is only legitimate when no established alternatives are available that could achieve the same ends.

This paper has investigated possible alternatives to germline modification as well as the (dis)advantages of GGE over existing reproductive technologies. Considering the extended applicability, it was shown that there are scenarios where GGE would provide the only option for intended parents to have healthy, biologically related offspring. Even though these constellations are comparably small in terms of numbers, they cannot be ignored. Following the recommendations from the NASEM and others, research and development of GGE should then be an option in these cases.

With regard to the argument of embryo protection, it was indicated that GGE has no significant moral advantages over embryo selection. Rather than replacing PGD, in most cases GGE would most likely be a supplemental tool and routinely create additional reasons for genetic testing and embryo selection. The argument of benefit and its underlying view on values was revealed as questionable. If accepted, it leads to the conclusion that the welfare of some future people is morally negligible. A possible modification of this view was not convincing either: it privileges GGE unduly, rendering pre‐emptive therapy preferable to the natural conception of a healthy child.

In light of all the above discussions, this paper does not support the strong claim that GGE is always or most often the better strategy than selective reproduction. However, the paper has provided further evidence that some research and development of GGE is morally justified. Despite the fact that modification of the human germline touches a wide variety of moral and social challenges that were not discussed in this paper, a case in favour of GGE can be made.

## CONFLICT OF INTEREST

The author declares no conflict of interest.

